# Efficient estimation of phase response curves via compressive sensing

**DOI:** 10.1186/1471-2202-12-S1-P61

**Published:** 2011-07-18

**Authors:** Sungho Hong, Erik De Schutter

**Affiliations:** 1 Computational Neuroscience Unit, Okinawa Institute of Science and Technology, Okinawa 904-0411, Japan; 2 Theoretical Neurobiology, University of Antwerp, B-2610 Antwerpen, Belgium

## 

The phase-response curve (PRC), relating the phase shift of the oscillator and externally given perturbation [1], is one of the most important tools for characterizing the biological oscillators, such as neurons, and studying their synchronization in a network [2]. In experiments, the PRC can be estimated by measuring the phase changes with given stimuli, and several methods have been proposed (reviewed in [3]). One of the problems in such estimation is that the timescales of a PRC, naturally inherited from neuronal dynamics, are around the range of interspike intervals, while the external stimuli, such as pulses or continuous noise, usually have strong power in higher frequency ranges. This problem has been addressed in many ways such as simple averaging [4], randomly selecting low frequency Fourier modes [2,3], assuming smooth priors [5], etc.

Here we propose a systematic and efficient approach of estimating the PRC based on the recently developed method in signal processing called Compressive Sensing (CS) [6]. CS is a framework for estimation of sparsely constructed signals from random observations, and therefore suitable for discovering a small number of low frequency Fourier modes composing the PRC from the experimental measurements with a much wider frequency spectrum.

**Figure 1:**
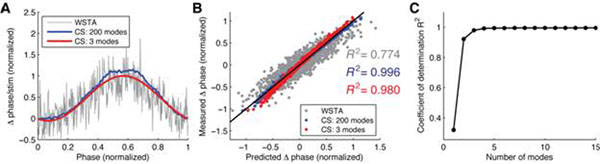
**A.** PRCs reconstructed from simulated data of the Morris-Lecar model via WSTA [4] and our CS-based method. The number of spikes was 400. In the CS case, the estimated PRCs composed of 200 (blue) and 3 (red) Fourier modes are shown. **B.** Predicted phase shifts vs measured phase shifts in each method. The black line represents the perfect match. **C.** Number of modes vs goodness-of-fit (*R*^2^) in the CS-based method.

Using simulated and experimental data, we show that our CS-based method can produce a decent estimate of the PRCs particularly when the number of spikes is so small that averaging cannot help (Fig. 1A, B). Furthermore, since contribution of each mode is systematically evaluated, one can also examine the tradeoffs between the number of modes and goodness-of-fit (Fig. 1C), and therefore pick out most relevant and predictive part of the measurements.
